# Morphological variability of wild-growing crown imperial (*Fritillaria imperialis* L.) germplasm in central region of Iran—implications for *in-situ* conservation initiatives

**DOI:** 10.1186/s12870-022-04032-7

**Published:** 2023-01-06

**Authors:** Mohammad Moradi, Alireza Khaleghi, Ali Khadivi

**Affiliations:** Department of Horticultural Sciences, Faculty of Agriculture and Natural Resources, Arak University, Arak, 38156-8-8349 Iran

**Keywords:** Conservation, Morphological variation, Overcollection, Superior specimens, Wild crown imperial

## Abstract

**Background:**

Crown imperial (*Fritillaria imperialis* L.) is a threatened bulbous plant which has great ornamental and medicinal values and importance. In the present study, a total of 100 specimens of wild-growing *F. imperialis* from 10 natural areas of Markazi province, Iran, representing one of the main centers of genetic diversity of this species, were evaluated using 37 phenotypic attributes during April 2021.

**Results:**

High level of genetic variation within populations (75%) and low levels of genetic variation among populations (25%) was revealed. The highest coefficient of variation (CV) was found in leaf trichome (82.00%) and then margin of crown leaves (80.44%). In addition, flower color (CV = 50.86%), flower number (CV = 44.61%), peduncle diameter (CV = 33.44%), and plant length (CV = 32.55%)—all important from an ornamental point of view- showed relatively high CV values. The CV was the lowest for flower shape, filament color, bulb shape, bulblet number, and floral scent. Ward cluster analysis identified two main clusters, containing 14 and 86 specimens, respectively. The first group consisted mainly of specimens from the adjacent Shahbaz and Rasvand populations. According to the principal component analysis (PCA), the first six components of data accounted for 88.36% of total variance. The Shahbaz-1, Shahbaz-2, Shahbaz-6, Shahbaz-7, Shahbaz-9, and Bolagh-8 specimens showed the highest variation and were separated from others, which they can be used further in breeding programs, while Sarchal-2, Bolagh-3, and Chepeqli-4 specimens showed the lowest variability. Moreover, the studied populations were clustered into four distinct groups, each including populations that were geographically close to one another.

**Conclusions:**

Although the examined specimens revealed high genetic diversity herein, the results indicated that wild-growing populations of *F. imperialis* are still at risk suffering from overcollection in the most of studied areas, especially in Deh-Sad and Tureh.

## Background

Genetic diversity refers to the aggregate of genetic variation within and between individuals and populations, and it is one of the main factors of concern in biodiversity conservation and plant breeding programs [1, 2]. Thus, the characterization of patterns of genetic diversity within a plant species is required for biodiversity conservation, management policies, and plant breeding [3]. Commonly, genetic diversity of plant species depends on different factors, such as breeding system, genetic drift, geographical, and ecological effects [4]. Therefore, the response of every species to environmental stress depends on its genetic diversity, and low genetic variability may decrease its ability to adapt to environmental stressors, thus affecting the long-term survival of the species [1, 5]. Genetic markers are fundamental for furnishing information about the genetic structure of populations, the rate of diversity within and among species, thus informing conservation management and policy [3]. One of the methods of genetic diversity assessment is morphological characterizations. Morphological characteristics are one of the commonly used tools which are applied in phenotypic evaluation, classifying the focal germplasm’s diversity for collection management, and selection of desired parents in targeted breeding schemes [2]. Morphological characterizations have been widely used to determine the genetic diversity of plant populations such as *Prunus cerasus *L. [6], *Juglans regia *L. [7], *Rosa* x *damascena *Mill. [8, 9], *Punica granatum *L. [2] and *Tulipa *spp. [10].

*Fritillaria* (Lilliaceae) is one of the most important genera of geophytes comprising about 140 perennial herbaceous species, which are mainly distributed in the temperate regions of the northern hemisphere [1, 11]. In Flora Iranica, 17 *Fritillaria* species have been reported in Iran, most of which are endemic to the country [12, 13]. Accordingly, Iran might be one of the main centers of genetic diversity of the genus *Fritillaria*. The species of *Fritillaria* have great medicinal and ornamental value and importance. From medicinal point of view, members of this genus are a botanical source of diverse medicinally active components, which have been commonly used in traditional Chinese, Turkish, Japanese, Pakistani, and South East Asian medicine for thousands of years [14, 15]. To date, over 150 different compounds, including steroidal alkaloids, saponins, glycosides, terpenoids, and many other medicinal compounds were discovered in various *Fritillaria* species [14–16]. *Fritillaria* members are also widely appreciated as ornamental plants and floriculture is an important industry with estimated worldwide production value over €60 billion [17]. Due to attractive flowers and high variation in their morphological and physiological traits, many members of the genus *Fritillaria* have a high potential for use as ornamental plants. Consequently, many new ornamental plants can be developed via artificial selection and hybridization breeding techniques.

The *Fritillaria imperialis* L. (crown imperial) is a perennial bulbous plant with high ornamental and medicinal importance [5]. Due to its orange-red downward facing large flowers as well as its crown of glossy green leaves at the top of stem, *F. imperialis* is a very attractive and valuable horticultural species [18]. This Iranian native plant is wild-growing at high altitudes of the alpine Zagros region [5]. Unfortunately, the wild-growing populations of this species are at risk of rapid extinction in Iran due to lack of protecting rules, overcollection, overexploitation, overgrazing, change of land uses (from pastures to farmlands), habitat fragmentation, no commercial cultivation, environmental changes, and pesticides [5, 19]. *F. imperialis* has a native distribution range from East-Central and South-Ease Turkey to West Himalayas [20]. Since, one of the major centers of genetic variation of *F. imperialis* is Iran within this range; its genetic assessment is required for effective conservation of this species. However, there are very few reports on the genetic diversity of these germplasm resources in Iran. Thus, in the present study, the assessment of genetic diversity of wild-growing *F. imperialis* is attempted, employing 100 specimens from 10 geographical areas of central region Iran collected; for these samples, phenotypic diversity within and among the populations was evaluated based on qualitative and quantitative attributes. The main objectives of this study were as follows: (1) to quantify the rate of morphological variation within and among populations studied, (2) to detect correlations between morphological variation and geographical distances among populations, (3) to develop recommendations for in situ conservation of this valuable species, (4) to recognize the most useful variables for discrimination within the examined specimens, and (5) to detect the specimens which might be useful as genetic resources for future breeding programs. This is the first study on genetic variation within and among population of *F. imperialis* in Markazi province, a central region of Iran.

## Material and methods

### Plant material

During April 2021, a total of 100 wild individual specimens of *F. imperialis* were studied from ten natural areas of Markazi province, a central region of Iran. A voucher specimen of this material has been deposited in the publicly available herbarium of Faculty of Agriculture and Natural Resources, Arak University, Iran with deposition number of FI-2342. The collection of plant was permitted from Agricultural and Natural Resources, Iran. The minimum distance between the specimens collected in each area was 20 m.

### Experimental site

The studied areas have high plant diversity, so these areas were selected based on the presence and potential diversity of *F. imperialis*. Geographical distribution and sampling information of the areas collected are presented in Table 1. Also, climatic data of the experimental areas in the growing season of 2021, calculated from the data between 01 and 30 April is shown in Table 2.

**Table 1:** Geographical distribution and sampling information of wild-growing *Fritillaria imperialis* populations collected from Markazi province, Iran

No	Collection site	Abbreviation	Latitude (N)	Longitude (E)	Altitude (m)	Annual precipitation (mm)	Soil texture
1	Shahbaz	Shah	33°53′	49°34′	2392–2548	490	Sandy loam
2	Rasvand	Rasv	33°54′	49°21′	2161–2249	450	Sandy clay loam
3	Surane	Sur	33°50′	49°27′	2346–2718	412	Sandy loam
4	Dastjerdeh	Dast	33°53′	49°25′	2151–2612	354	Silty clay
5	Tureh	Ture	34°02′	49°15′	1920–1996	224	Sandy clay
6	Deh-Sad	Sad	34°12′	49°22′	1874–1965	223	Clay loam
7	Eyvand-e-Now	Eyva	34°13′	49°25′	2194–2270	213	Sandy clay
8	Sarchal	Sarch	34°10′	49°06′	2362–2474	260	Silty clay
9	Bolagh	Bola	33°59′	49°21′	1972–2060	390	Sandy clay loam
10	Chepeqli	Chep	33°58′	49°33′	2474–2650	374	Sandy clay loam

**Table 2:** Climatic data of the experimental areas in the growing season of 2021, calculated from the data between 01 and 30 April

No	Collection site	Mean minimum temperature (˚C)	Mean maximum temperature (˚C)	Mean temperature (˚C)	Mean relative humidity (%)	Total precipitation (mm)
1	Shahbaz	4.4	20.7	15.2	38.5	15.8
2	Rasvand	4.8	21.8	15.9	36.8	12.6
3	Surane	5.1	22.1	16.2	33.5	11.4
4	Dastjerdeh	5.6	23.6	17.2	34.8	7.8
5	Tureh	6.1	23.2	17.3	27.3	8.2
6	Deh-Sad	6.8	23.7	17.9	26.6	3.2
7	Eyvand-e-Now	6.9	23.8	30	27.8	4.1
8	Sarchal	6.2	23.1	17.3	31.2	4.9
9	Bolagh	5.3	22.4	16.5	33.6	10.3
10	Chepeqli	5.9	22.9	17.1	34.8	9.8

### Data observation

All morphological traits were measured and recorded at full flowering time. Thirty-seven phenotypic attributes, including qualitative and quantitative traits of flower, stem, leaf, and bulb, were used for the assessment of the genetic variability of the studied specimens. Quantitative traits, such as plant length, length and diameter of peduncle, width and length of leaf, width and length of flower, bulb diameter, and bulb length were measured using a digital caliper. In addition, qualitative traits were surveyed based on rating, scoring, and coding.

### Statistical analysis

Analysis of variance (ANOVA) was performed to evaluate the variation among specimens based on the traits measured using SAS software [21]. Simple correlations between traits were determined using Pearson correlation coefficients [22]. Principal component analysis (PCA) was used to investigate the relationship between specimens and determine the main traits that were effective in specimens segregation using SPSS software. Hierarchical cluster analysis (HCA) was performed using Ward’s method and Euclidean coefficient using PAST software [23]. The first and second principal components (PC1/PC2) were used to create a scatter plot with PAST software [23].

## Results and discussion

### Genetic diversity

**Fig. 1:**
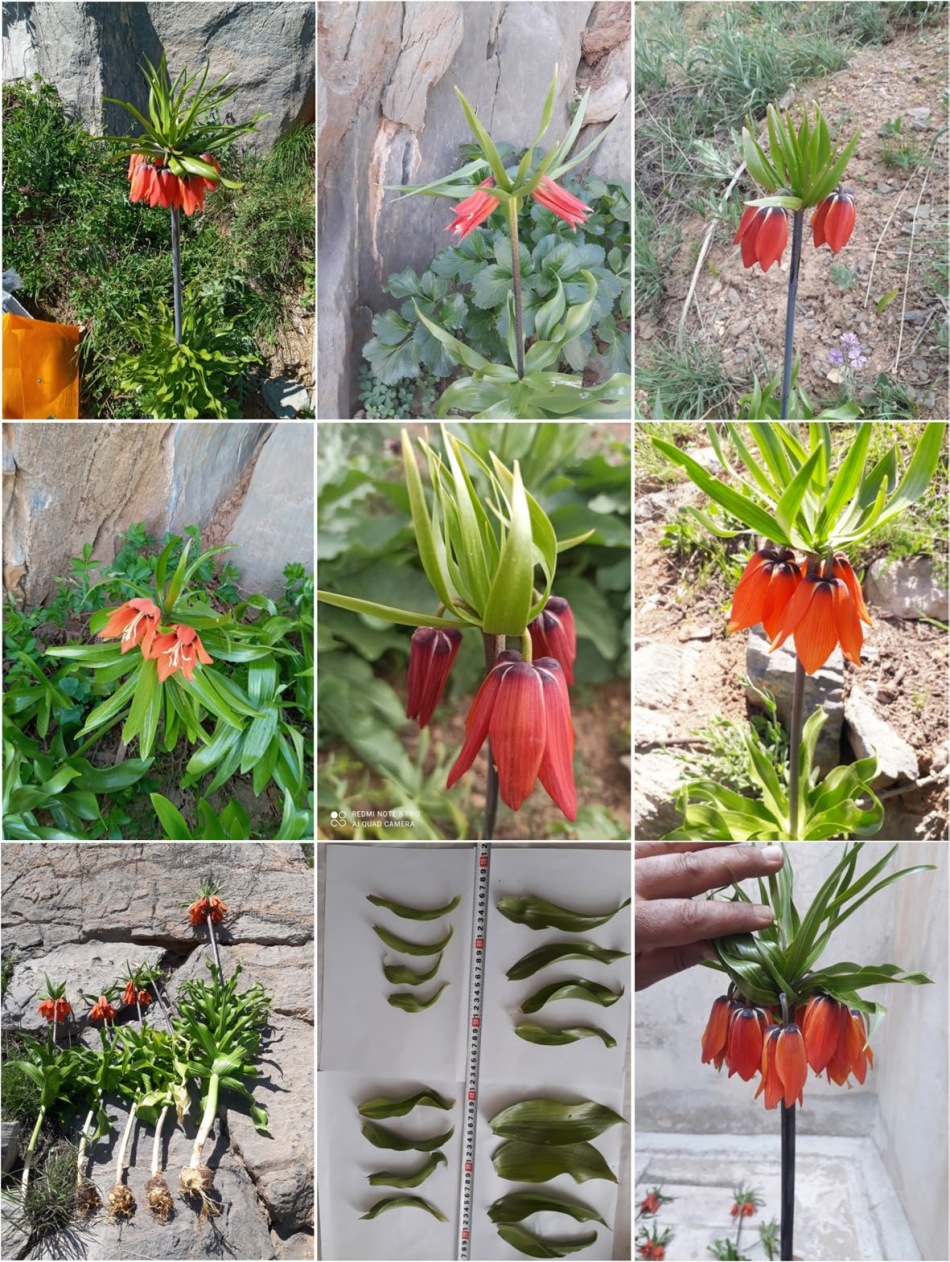
Variation of morphological characteristics, such as flower color, flower number, peduncle length, and leaf size of wild-growing *Fritillaria imperialis* from Markazi province, Iran

Analysis of variance (ANOVA) showed significant difference among the populations studied based on the measured characteristics (Table 3). In agreement with the present results, Mucciarelli et al. [3] observed high phenotypic variation within populations of *F. tubiformis* var. *burnatii* (Planch.) Rouy. Also, high levels of genetic diversity were reported among seven populations of *F. imperialis* in the Zagros region of Iran [5]. This high variability within populations has been ascribed to the outcrossing mating system [4, 24]. High coefficients of variation (CV) were detected in most of the measured traits that indicated significant variations within populations. The highest CVs were found in leaf trichome (82.00%) and margin of crown leaves (80.44%). Also, flower color (50.86%), flower number (44.61%), peduncle diameter (33.44%), and plant length (32.55%)- all important from an ornamental point of view, showed relatively high CV values (Table 4; Fig. 1). In contrast, flower shape, filament color, bulb shape, bulblet number, and floral scent did not show differences among the specimens. Morphological attributes with lower CVs were more homogeneous and can be considered as stable characters among specimens, while the characteristics with higher variation than 20.00% were more distinct between specimens and can be reliable markers for differentiating specimens [25]. In other words, the traits with higher amplitude of variation are more suitable for selection in breeding programs.

**Table 3:** ANOVA summary for wild-growing *Fritillaria imperialis* populations from Markazi province, Iran

Sources of changes	DF	Mean Square											
		Plant length	Peduncle length	Peduncle diameter	Peduncle diameter under flower	Leaf number	Bottommost leaf length	Bottommost leaf width	Second bottommost leaf length		Second bottommost leaf width	Flower diameter	
Population	9	2265.26^**^	365.14^**^	40.06^**^	30.50^**^	420.09^**^	41.18^**^	18.45^**^	35.51^**^		15.47^**^	65.91^**^	
Error	90	159.01	27.67	4.08	3.46	50.53	2.05	0.78	2.51		0.86	4.39	
CV (%)	-	21.92	18.64	24.90	24.73	29.98	10.15	15.65	12.37		23.33	7.41	
Sources of changes	DF	Mean Square											
		Flower length	Underground stem length	Underground stem diameter	Bulb diameter	Bulb length	Width of largest crown leaf	Length of largest crown leaf		Number of crown leaves	Flower Number	Pedicel length	Pedicel diameter
Population	9	81.29^**^	53.02^**^	8.24^**^	136.09^**^	325.71^**^	1.07^**^	0.63^**^		337.09^**^	15.68^**^	4.39^ ns^	0.76^**^
Error	90	5.71	3.98	0.80	23.20	38.19	0.07	0.05		20.63	1.23	2.77	0.05
CV (%)	-	8.00	13.03	7.28	13.37	16.22	1.20	2.74		24.47	31.04	5.20	9.02

^ns^nonsignificant

* *p* < 0.05., ** *p* < 0.01

**Table 4:** The minimum, maximum, mean, standard deviation, and coefficient of variation of morphological traits measured in the studied specimens of wild-growing *Fritillaria imperialis* from Markazi province, Iran

No	Character	Unit	Min	Max	Mean	SD	CV (%)
1	Plant length	cm	31	106	57.52	18.72	32.55
2	Peduncle length	cm	17	46	28.21	7.64	27.08
3	Peduncle diameter	mm	5.16	14.46	8.11	2.71	33.44
4	Peduncle diameter under flower	mm	4.19	13.37	7.52	2.43	32.35
5	Peduncle color	Code	1	5	3.00	0.90	29.97
6	Leaf number	Number	15	48	23.71	9.17	38.68
7	Leaf color	Code	1	5	3.20	1.41	43.97
8	Leaf margin	Code	1	3	1.40	0.80	57.43
9	Leaf shape	Code	1	7	4.40	1.57	35.68
10	Bottommost leaf length	cm	10.00	20.00	14.11	2.37	16.79
11	Bottommost leaf width	cm	2.85	8.50	5.66	1.55	27.33
12	Second bottommost leaf length	cm	4.80	19.00	12.81	2.35	18.33
13	Second bottommost leaf width	cm	2.00	7.50	3.97	1.48	37.25
14	Flower diameter	mm	22.20	32.80	28.30	3.16	11.17
15	Flower length	mm	26.14	34.80	29.86	3.55	11.88
16	Anther color	Code	1	3	2.80	0.60	21.54
17	Flower color	Code	1	9	5.00	2.54	50.86
18	Underground stem length	cm	10.12	26.00	15.31	2.91	18.97
19	Underground stem diameter	mm	10.88	16.95	12.34	1.22	9.87
20	Bulb diameter	mm	32.15	57.42	36.02	5.79	16.06
21	Bulb length	mm	33.15	65.16	38.09	8.02	21.06
22	Leaf trichome	Code	0	1	0.60	0.49	82.00
23	Stem trichome	Code	1	5	3.00	0.90	29.97
24	Width of largest crown leaf	mm	22.00	23.40	22.56	0.41	1.80
25	Length of largest crown leaf	cm	7.90	9.30	8.28	0.32	3.91
26	Number of crown leaves	Number	8	44	18.56	7.03	37.87
27	Shape of crown leaves	Code	1	7	4.00	2.42	60.50
28	Color of crown leaves	Code	1	5	2.60	1.50	57.85
29	Margin of crown leaves	Code	1	5	1.60	1.29	80.44
30	Flower Number	Number	2	8	3.58	1.60	44.61
31	Pedicel length	mm	22.40	33.15	31.98	1.71	5.34
32	Pedicel diameter	mm	2.10	3.50	2.52	0.34	13.57

From an ornamental point of view, plant length, peduncle length, peduncle diameter, flower color, flower number, flower diameter, and flower length are very important defining largely the floricultural value of many species. In the present study, moderate to high genetic variations were observed in each of the above-mentioned traits. Plant length varied from 31.00 to 106.00 cm with an average of 57.52 cm, while peduncle length ranged from 17.00 to 46.00 cm. The highest plant length and peduncle length were observed in the Shahbaz-6 specimen and the lowest in the Eyvand-e-Now-10 specimen. Also, the highest peduncle diameter in downward and upward was detected in the Shahbaz-6 specimen; and the lowest peduncle diameter was detected in the Eyvand-e-Now-5 specimen. A high variation was found among specimens regarding flower number and flower color. The flower number varied from 2 to 8, and flower color varied from light orange to red. In most of the specimens, the petal was orange or dark orange (60 specimens) (Table 5). The highest flower number was observed in specimens of Shahbaz population, including Shahbaz-2, Shahbaz-6, and Shahbaz-9 specimens. Flower diameter varied from 22.20 (in Deh-Sad-7 specimen) to 32.80 mm (in Shahbaz-6 specimen) with CV of 11.17%, and flower length ranged from 26.14 (in Shahbaz-6 specimen) to 34.80 mm (in Eyvand-e-Now-10 specimen) with CV of 11.88%.

**Table 5:** Frequency distribution for the measured qualitative morphological traits in the studied specimens of wild-growing *Fritillaria imperialis* from Markazi province, Iran

Qualitative trait	Frequency (no. of specimens)					
	0	1	3	5	7	9
Peduncle color	-	Grayish-green (10)	Gray (80)	Dark grey (10)	-	-
Leaf color	-	Light green (20)	Green (50)	Dark green (30)	-	-
Leaf margin	-	Smooth (80)	Little wavy (20)	-	-	-
Leaf shape	-	Narrow and elongated (10)	Broad and elongated (20)	Broad and slightly elongated (60)	Broad (10)	-
Anther color	-	Light yellow (10)	Yellow (90)		-	-
Petal color	-	Light orange (10)	Orange (30)	Dark orange (30)	Light red (10)	Red (20)
Leaf trichome	Absent (40)	Very little (60)	-	-	-	-
Stem trichome	-	Little (10)	Moderate (80)	High (10)	-	-
Shape of crown leaves	-	Narrow and elongated (30)	Elongated (20)	Slightly broad and elongated (20)	Broad and elongated (30)	-
Color of crown leaves	-	Light green (40)	Green (40)	Dark green (20)	-	-
Margin of crown leaves	-	Smooth (80)	Little wavy (10)	Wavy (10)	-	-

Peduncle color was gray in most of the specimens (90), while it was dark gray in all specimens of Tureh population. Also, anther color was yellow in 90 specimens, while it was light yellow in 10 specimens (Table 5). Leaf color of 50 specimens was green. The length of the bottommost leaf was from 10.00 to 20.00 cm, while the width of the bottommost leaf varied from 2.85 to 8.50 cm. The highest leaf number (48 leaves) was observed in Shahbaz-6 specimen and the lowest one in Bolagh-1 specimen (Table 4). In most of the specimens, the leaf margin and leaf shape were smooth (80 specimens) and broad-slightly elongated (60), respectively (Table 5). Bulb diameter varied from 32.15–57.42 mm, while bulb length was from 33.15 to 65.16 mm (Table 4). Most probably, the high genetic diversity has a positive effect on the long-term persistence of this species by increasing the ability of plant individuals to adapt to changing environmental conditions, and accordingly, decreased genetic variation would affect population viability by reducing individual fitness [24].

### Principal component analysis

Principal component analysis (PCA) was used to identify patterns of variability among the specimens studied. This method is one of the mighty multivariate statistical techniques to classify the assessed characteristics into effective groups. In this method, traits are placed in the components, each containing different characters. The PCA method can reveal the major differences among the specimens surveyed and also may decrease the amount of data. The relative variance of each component indicates the importance of the component in the variance of the studied traits and is expressed as percentage. The first component justifies the greatest amount of variance, and the subsequent factors justify the remaining changes after the first component [26]. This analysis had been previously used to assess germplasm of different socioeconomically valuable species, such as *Crocus sativus *L. [27], *Triticum monococcum* L. subsp.* aegilopoides*(Link) Thell. [28], *Allium sativum *L. [29], *Juglans regia* [30], *Corchorus olitorius* L. [31], and *Emmenopterys henryi* Oliv. [32].

For each factor, the loading of the principal component was considered to be more than 0.63, indicating that the first six components of data accounted for 88.36% of total variance (Table 6). The first component (PC1), included 17 variables and explained 48.95% of total variance, showing that these characteristics had most of the diversity among specimens and presented the greatest effect on the differentiation of specimens. The PC1 had the most positive relationship with plant length, peduncle length and diameter, leaf number, leaf length, and width, flower diameter and length, length and diameter of underground stem, diameter and length of bulb, length of largest crown leaf, number of crown leaves, and pedicel diameter. The PC2 was associated with five characteristics, including leaf color, leaf margin, trichome of stem, color of crown leaves, and margin of crown leaves, accounting for 15.42% of the total variation. The PC3, which explained 7.53% of the total variation, had a negative relationship with shape of crown leaves and was positively correlated with leaf trichome and second bottommost leaf width.

**Table 6:** Eigenvectors of six principal component axes from PCA analysis of morphological variables in the studied specimens of wild-growing *Fritillaria imperialis* from Markazi province, Iran

Characteristic	PC1	PC2	PC3	PC4	PC5	PC6
Plant length	**0.99**	0.13	-0.01	0.03	0.00	0.03
Peduncle length	**0.95**	0.11	-0.12	-0.01	-0.04	-0.13
Peduncle diameter	**0.93**	0.15	-0.10	-0.01	-0.14	0.02
Peduncle diameter under flower	**0.95**	0.09	0.03	0.05	-0.01	-0.01
Peduncle color	0.25	-0.49	0.51	-0.11	-0.61	-0.06
Leaf number	**0.91**	0.20	-0.12	-0.07	0.03	0.17
Leaf color	-0.09	**0.77**	-0.22	-0.48	-0.26	0.14
Leaf margin	0.06	**0.95**	0.02	0.07	0.20	0.01
Leaf shape	0.00	0.02	0.04	0.18	**0.95**	0.03
Bottommost leaf length	**0.94**	0.15	-0.04	0.09	-0.14	-0.02
Bottommost leaf width	**0.88**	-0.05	0.24	0.07	0.16	-0.22
Second bottommost leaf length	**0.92**	0.09	0.05	0.00	-0.11	-0.03
Second bottommost leaf width	0.56	-0.24	**0.63**	0.13	0.25	0.03
Flower diameter	**0.86**	0.02	-0.05	0.01	-0.11	-0.35
Flower length	**0.85**	0.01	-0.06	-0.01	-0.13	-0.37
Anther color	0.31	0.06	0.07	**0.74**	0.13	-0.23
Flower color	-0.38	-0.04	-0.17	**0.81**	0.09	0.15
Underground stem length	**0.90**	0.05	0.23	0.04	0.15	-0.03
Underground stem diameter	**0.91**	0.10	0.11	-0.05	0.12	0.12
Bulb diameter	**0.78**	0.09	-0.17	-0.02	0.03	0.30
Bulb length	**0.85**	0.24	-0.02	-0.08	0.10	0.33
Leaf trichome	-0.12	0.35	**0.65**	0.62	0.09	0.08
Stem trichome	0.32	**0.64**	0.44	-0.05	-0.02	0.00
Width of largest crown leaf	0.49	0.60	-0.26	0.38	0.08	0.17
Length of largest crown leaf	**0.95**	0.21	0.00	-0.03	0.02	0.13
Number of crown leaves	**0.95**	0.13	0.14	0.01	0.03	0.04
Shape of crown leaves	0.28	-0.06	**-0.82**	0.11	0.12	-0.21
Color of crown leaves	0.19	**0.89**	-0.03	0.22	-0.20	-0.02
Margin of crown leaves	0.25	**0.90**	0.19	-0.08	0.18	-0.04
Flower Number	0.59	0.61	-0.22	0.08	0.13	0.30
Pedicel length	0.03	0.05	0.15	-0.03	0.02	**0.73**
Pedicel diameter	**0.96**	0.14	0.01	0.03	-0.01	-0.06
Total	15.67	4.94	2.41	2.14	1.73	1.39
% of Variance	48.95	15.42	7.53	6.69	5.42	4.34
Cumulative % of variance	48.95	64.38	71.9	78.6	84.02	88.36

Bold values indicate the characteristic that most influence each PC

### Cluster analysis

**Fig. 2:**
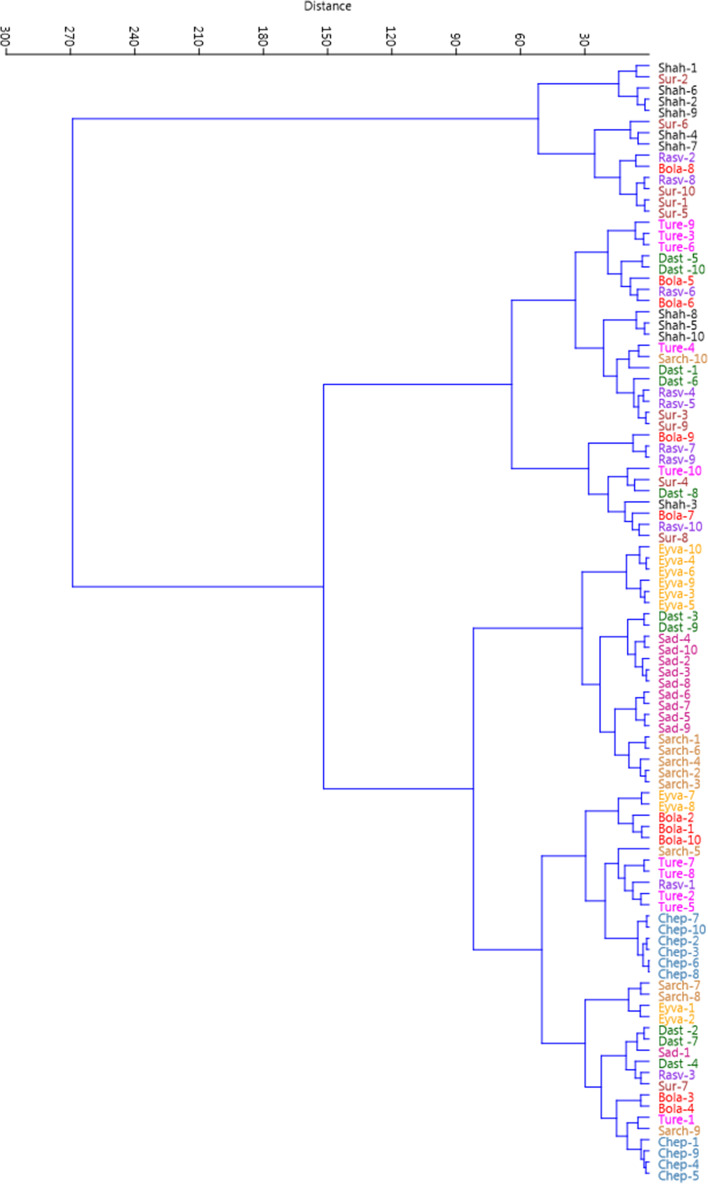
Ward dendrogram of cluster analysis for the studied specimens of wild-growing *Fritillaria imperialis* from Markazi province, Iran based on morphological characters (for an explanation of specimen symbols, see Table 1)

The Ward dendrogram which was based on all the variables measured, indicated resemblances and heterogeneities among the specimens [2]. Ward cluster analysis identified two main clusters, containing 14 and 86 specimens in each (Fig. 2). The first group (I) consisted mainly of specimens from the adjacent Shahbaz and Rasvand populations (small geographical distance from each other). The second group (II) formed two sub-clusters. The sub-cluster II-A included 29 specimens, while the sub-clusters II-B included 57 specimens; including all the specimens of the Eyvand-e-Now, Deh-Sad and Chepeqli populations. The specimens that were placed in cluster I were characterized by the highest values of plant length, peduncle length, peduncle diameter, flower diameter, and leaf number.

### Phenotypic variation

**Fig. 3:**
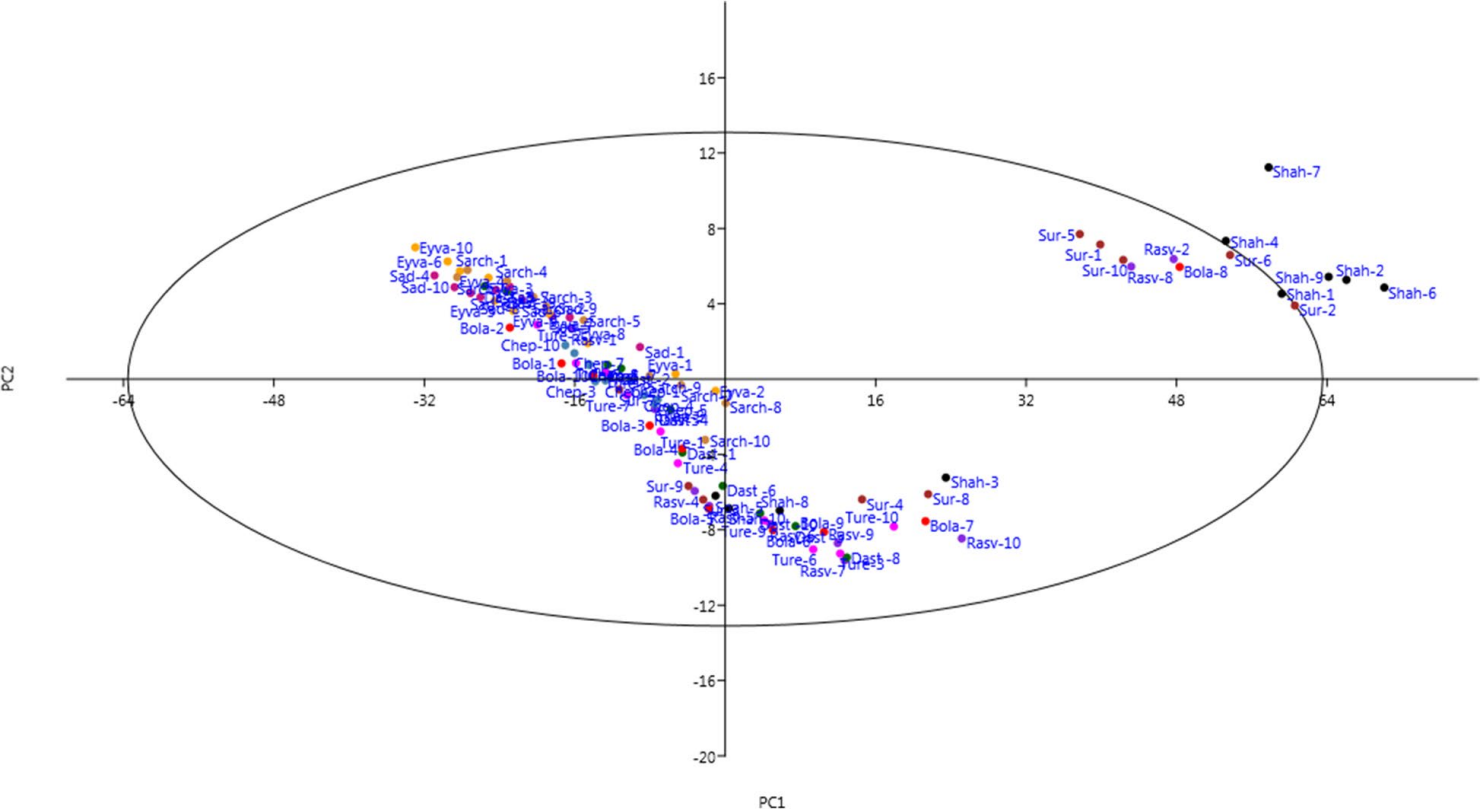
Two-dimensional bi-plot for PC1/PC2 (64.38% of total variance) among the studied specimens of wild-growing *Fritillaria imperialis* from Markazi province, Iran (for an explanation of specimen symbols, see Table 1)

A scatter plot was created based on the PC1 and PC2 (64.38% of total variance) which resounded relationship among the specimens in terms of morphological characteristics and phenotypic resemblance (Fig. 3). Based on results of the scatter plot, the superior specimens, including Shahbaz-1, Shahbaz-2, Shahbaz-4, Shahbaz-6, Shahbaz-7, Shahbaz-9, Rasvand-2, Rasvand-8, Surane-1, Surane-2, Surane-5, Surane-6, Surane-10, andBolagh-8, were separated from others, due to the highest values of plant length, peduncle length, peduncle diameter, flower diameter, and leaf number. These results were consistent with the results of Ward dendrogram.

**Fig. 4:**
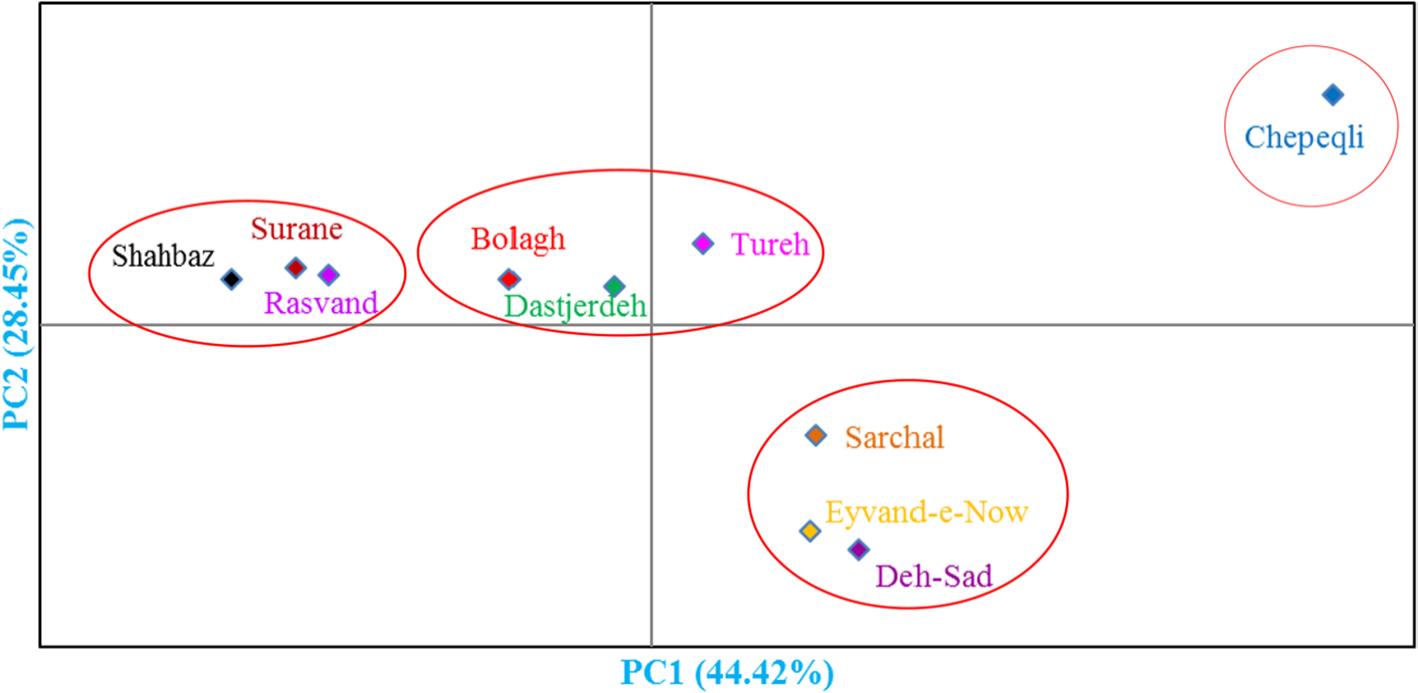
Bi-plot for 10 Iranian populations studied of wild-growing *Fritillaria imperialis* based on the morphological traits

Based on the population analysis (Fig. 4), the studied areas were discerned into four distinct groups with the populations placed in each group being geographically close to one another. The Surane, Shahbaz, and Rasvand populations were placed into the first group and were characterized by high values in plant length, peduncle length, peduncle diameter, flower diameter and length, leaf number, flower number and diameter, and length of bulb, while Bolagh, Dastjerdeh, and Tureh populations formed the second group and were characterized by moderate values in plant length, peduncle length, peduncle diameter, leaf number, flower diameter and length, and diameter, and length of bulb. In addition, Eyvand-e-Now, Deh-Sad, and Sarchal populations were placed into the third group and were characterized by low values in plant length, peduncle length, diameter and length of flower, bulb diameter, and bulb length. Finally, Chepeqli population formed the fourth group and was characterized by low values in peduncle diameter, leaf number, and number of flower. The results of bi-plot of the populations clearly showed that grouping has resulted from geographic location. Moreover, this grouping of populations may have been influenced by geographical barriers and ecological factors. The pairwise population matrix showed that the highest genetic distance was between Shahbaz and Ture populations (D = 29/55), while the lowest genetic distance was between Surane and Rasvand populations (D = 21/75) (Table 7). These results were consistent with the geographical distance of the populations. The lowest geographical distance was between Surane and Rasvand populations. It is proven that geographic distribution is closely associated with genetic variation within and among populations [24].

**Table 7:** Pairwise population matrix of binary genetic distance of wild-growing *Fritillaria imperialis* from Markazi province, Iran

Population	Shahbaz	Rasvand	Surane	Dastjerdeh	Tureh	Deh-Sad	Eyvand-e-Now	Sarchal	Bolagh	Chepeqli
Shahbaz	20.02	_	_	_	_	_	_	_	_	_
Rasvand	29.10	19.64	_	_	_	_	_	_	_	_
Surane	28.11	21.75	20.15	_	_	_	_	_	_	_
Dastjerdeh	28.47	23.59	24.15	19.75	_	_	_	_	_	_
Tureh	29.55	24.82	24.23	26.66	19.42	_	_	_	_	_
Deh-Sad	28.73	24.07	26.45	24.83	27.92	19.31	_	_	_	_
Eyvand-e-Now	25.68	29.39	28.62	28.39	28.34	28.67	19.68	_	_	_
Sarchal	27.59	24.07	23.48	24.75	22.88	26.33	27.08	19.75	_	_
Bolagh	27.21	24.44	24.09	24.58	25.52	25.93	26.35	23.81	19.86	_
Chepeqli	28.77	26.3	25.55	25.84	23.66	25.95	28.13	23.81	23.00	17.97

The highest value of Shannon index was related to Chepeqli population, while the highest values of Menhinick and Margalef indices were related to Deh-Sad population (Table 8). The Shannon index is an information statistic index, which means it assumes all species are represented in a sample and that they are randomly sampled [25].

**Table 8:** Diversity indices of wild-growing *Fritillaria imperialis* populations from Markazi province, Iran

Population	Shannon_H	Menhinick	Margalef
Shahbaz	2.78	0.91	3.20
Rasvand	2.79	1.00	3.30
Surane	2.78	0.95	3.25
Dastjerdeh	2.79	1.06	3.36
Tureh	2.80	1.05	3.35
Deh-Sad	2.79	1.14	3.45
Eyvand-e-Now	2.80	1.13	3.43
Sarchal	2.79	1.10	3.41
Bolagh	2.80	1.03	3.33
Chepeqli	2.81	1.08	3.39
Min	2.78	0.91	3.20
Max	2.81	1.14	3.45

### Implications for conservation and sustainable utilization

The results herein showed that the population of the areas with higher altitude and more snowfall, such as Shahbaz population, had higher density and quality of specimens in respect to the populations of areas with lower altitude and less snowfall, including Deh-Sad and Tureh populations. The in situ observations showed that overgrazing and overcollection by local people is exercised widely in the areas with lower altitude. Furthermore, the amount of rainfall in these areas is less compared with other areas. Based on meteorological data, the highest annual rainfall is recorded every year in Shahbaz area, which had the highest superior specimens. Compared with other areas, it is also more difficult for local people to access the Shahbaz area for grazing and harvesting. According to our field visiting, it was observed that the size and density of local populations are rapidly decreasing due to overharvesting and overgrazing, especially in low altitudes. It is known that overcollecting and overgrazing may also lead to reduced genetic diversity in wild-growing populations [24]. Furthermore, the wild-growing populations of *F. imperialis* in some of the areas studied in this survey are currently at high risk of rapid eradication, including Alborj and Si-Qazghan areas (data not shown). In the Alborj area, the specimens were highly overgrazed before the flowering stage; and in Si-Qazghan area, due to the severe decrease in rainfall in 2021, the wild-growing stands of *F. imperialis* were found to be chlorosized in the vegetative stage and did not enter the flowering phase; therefore, no data were collected from these two populations. Commonly, it is expected that density and sizes of populations will be sharply reduced by overgrazing and overharvesting of the wild resources of *F. imperialis*. Consequently, this will inevitable result to patches and local extinction events, and decreased ability to adapt to changing environments. For instance, previous reports have shown that the genetic diversity of the American ginseng has significantly decreased by random harvesting [19, 24, 33]. Moreover, decrease of populations size and number of populations due to overharvesting and overgrazing is reported in *Thymus algeriensis*, with populations from the arid zone being the most affected ones [34]. Based on the UNEP climate classification, most parts of Iran and especially the central regions have an arid and semi-arid climate [35, 36]. Therefore, the protection of scattered habitats of at least small size with small populations of *F. imperialis* naturally thriving in such semi-arid and arid lowland areas is of vital importance.

## Conclusions

Based on morphological data, high levels of genetic variation were detected within populations of *F. imperialis*, which would be useful as genetic resources for future breeding programs and creation of new *Fritillaria* varieties for ornamental purposes. The Shahbaz-1, Shahbaz-2, Shahbaz-6, Shahbaz-7, Shahbaz-9, and Bolagh-8 specimens showed the highest variation and were separated from others, which they can be used further in breeding programs, while Sarchal-2, Bolagh-3, and Chepeqli-4 specimens showed the lowest variability. From an ornamental point of view, the Shahbaz-1, Shahbaz-2, Shahbaz-6, Shahbaz-7, and Shahbaz-9, all specimens from Shahbaz area, seem to be the most promising for breeding programs, since they were characterized by high values of important ornamental features (plant length, peduncle length, peduncle diameter, flower number, flower size, and leaf number). Based on bi-plot of population analysis, the studied populations were discerned into four distinct groups, all correlated with observed geographical distances among populations (each group included adjacent populations). These results were confirmed by analysis of the pairwise population matrix of the mean binary genetic distance. Although the studied specimens have shown high genetic diversity in this study, our results and observations have shown that the wild-growing populations of *F. imperialis* are currently at risk of rapid depletion in the most of studied areas, especially in Deh-Sad, Tureh, Alborj, and Si-Qazghan. The main reasons for this trend were overgrazing, overharvesting, and climate change. Preservation of genetic diversity is one of the main objectives in conserving the threatened species. Therefore, the protection of existing wild-growing populations of *F. imperialis* in Iran is essential and vital. Limitation of overgrazing and overharvesting, surveillance and customs control are suggested to maintain effective population sizes of *F. imperialis* conserved in situ. Moreover, domestication, development of species-specific propagation protocols, and cultivation protocols prior to artificial selection of desired specimens, are effective measures to preserve this species allowing at the same time its sustainable exploitation in the ornamental sector.

## Data Availability

The data that support the findings of this study are available from the corresponding author upon reasonable request.
